# Increased prevalence of loneliness and associated risk factors during the COVID-19 pandemic: findings from the Canadian Longitudinal Study on Aging (CLSA)

**DOI:** 10.1186/s12889-023-15807-4

**Published:** 2023-05-12

**Authors:** Susan A. Kirkland, Lauren E. Griffith, Urun Erbas Oz, Mary Thompson, Andrew Wister, Laura Kadowaki, Nicole E. Basta, Jacqueline McMillan, Christina Wolfson, Parminder Raina, on behalf of the Canadian Longitudinal Study on Aging (CLSA) Team, Laura Anderson, Cynthia Balion, Andrew Costa, Yukiko Asada, Benoȋt Cossette, Melanie Levasseur, Scott Hofer, Theone Paterson, David Hogan, Teresa Liu-Ambrose, Verena Menec, Philip St. John, Gerald Mugford, Zhiwei Gao, Vanessa Taler, Patrick Davidson, Theodore Cosco

**Affiliations:** 1 Departments of Community Health & Epidemiology and Medicine, Dalhousie University, Halifax, NS Canada; 2 Department of Health Research Methods, Evidence, and Impact, McMaster University, 1280 Main St W. MIP-309A, Hamilton, ON L8S 4K1 Canada; 3 Labarge Centre for Mobility in Aging, McMaster University, Hamilton, ON Canada; 4 McMaster Institute for Research On Aging, McMaster University, Hamilton, ON Canada; 5 Department of Statistics and Actuarial Science, University of Waterloo, Waterloo, ON Canada; 6 Gerontology Research Centre, Simon Fraser University, British Columbia, Canada; 7 Department of Epidemiology, and Occupational Health, McGill University, BiostatisticsMontreal, QC Canada; 8 Division of Geriatric Medicine, Department of Medicine, Cumming School of Medicine, University of Calgary, Calgary, AB Canada; 9 Department of Medicine, McGill University, Montreal, QC Canada; 10 Research Institute of the McGill University Health Centre, Montreal, QC Canada

**Keywords:** CLSA, COVID-19, Pandemic, Loneliness, Longitudinal

## Abstract

**Background:**

Older adults have been disproportionately impacted by COVID-19 and related preventative measures undertaken during the pandemic. Given clear evidence of the relationship between loneliness and health outcomes, it is imperative to better understand if, and how, loneliness has changed for older adults during the COVID-19 pandemic, and whom it has impacted most.

**Method:**

We used “pre-pandemic” data collected between 2015–2018 (*n* = 44,817) and “during pandemic” data collected between Sept 29-Dec 29, 2020 (*n* = 24,114) from community-living older adults participating in the Canadian Longitudinal Study on Aging. Loneliness was measured using the 3-item UCLA Loneliness Scale. Weighted generalized estimating equations estimated the prevalence of loneliness pre-pandemic and during the pandemic. Lagged logistic regression models examined individual-level factors associated with loneliness during the pandemic.

**Results:**

We found the adjusted prevalence of loneliness increased to 50.5% (95% CI: 48.0%-53.1%) during the pandemic compared to 30.75% (95% CI: 28.72%-32.85%) pre-pandemic. Loneliness increased more for women (22.3% vs. 17.0%), those in urban areas (20.8% vs. 14.6%), and less for those 75 years and older (16.1% vs. 19.8% or more in all other age groups). Loneliness during the pandemic was strongly associated with pre-pandemic loneliness (aOR 4.87; 95% CI 4.49–5.28) and individual level sociodemographic factors [age < 55 vs. 75 + (aOR 1.41; CI 1.23–1.63), women (aOR 1.34; CI 1.25–1.43), and no post-secondary education vs. post-secondary education (aOR 0.73; CI 0.61–0.86)], living conditions [living alone (aOR 1.39; CI 1.27–1.52) and urban living (aOR 1.18; CI 1.07–1.30)], health status [depression (aOR 2.08; CI 1.88–2.30) and having two, or ≥ three chronic conditions (aOR 1.16; CI 1.03–1.31 and aOR 1.34; CI 1.20–1.50)], health behaviours [regular drinker vs. non-drinker (aOR 1.15; CI 1.04–1.28)], and pandemic-related factors [essential worker (aOR 0.77; CI 0.69–0.87), and spending less time alone than usual on weekdays (aOR 1.32; CI 1.19–1.46) and weekends (aOR 1.27; CI 1.14–1.41) compared to spending the same amount of time alone].

**Conclusions:**

As has been noted for various other outcomes, the pandemic did not impact all subgroups of the population in the same way with respect to loneliness. Our results suggest that public health measures aimed at reducing loneliness during a pandemic should incorporate multifactor interventions fostering positive health behaviours and consider targeting those at high risk for loneliness.

**Supplementary Information:**

The online version contains supplementary material available at 10.1186/s12889-023-15807-4.

## Background

Loneliness and social isolation are public health concerns that affect aging societies globally [1]. Whereas social isolation is defined as the objective state of having few social relationships or infrequent social contact with others, loneliness is the subjective feeling of not having one’s social needs met [2]. Thus, while social isolation and loneliness often occur together, it is possible to be socially isolated but not lonely, and to be lonely but not socially isolated. Social isolation is a known risk factor for loneliness, and in turn, both are risk factors for morbidity and mortality with a magnitude comparable to modifiable risk factors such as smoking, lack of exercise, obesity and high blood pressure [3, 4]. In addition, loneliness has been associated with decreased resistance to infection, cognitive decline, and mental health conditions such as depression and dementia [4]. Loneliness has also been associated with decreased quality of life and wellbeing [5]. Coyle et al. [6] found that loneliness was associated with 17% higher odds of having a mental health issue, and, although both loneliness and social isolation are associated with poorer mental health, Cornwell et al. [7] found that loneliness had a decidedly stronger association with mental health than social isolation. The growing public health and policy concern about loneliness is exemplified by the appointment of Ministers of Loneliness in the United Kingdom and Japan and the inclusion of addressing loneliness as one of the key pillars of the World Health Organization’s Decade of Healthy Ageing (2021–2030) [1]. In the current climate of a global pandemic where physical distancing, stay-at-home orders, and lockdowns have been implemented, concerns around loneliness have become even more salient.

Prior to the start of the coronavirus disease 2019 (COVID-19) pandemic, a meta-analysis of 31 studies including data from over 120,000 older people from 29 high income countries produced an estimate of the prevalence of loneliness of 28.5% (95%CI: 23.9%—33.2%) [8]. However, there was significant heterogeneity across studies with individual study estimates ranging from 11 to 55%, likely due to differences in the types of measurement tools used, methods of data collection and population characteristics. Regardless of the heterogeneity, over 60% of the studies indicated at least one in four older adults reported experiencing loneliness prior to the pandemic. Studies undertaken during the COVID-19 pandemic have demonstrated a significant increase in reported loneliness among older adults world-wide [9]. The most recent systematic review by Ernst et al. [10] included only longitudinal studies examining loneliness prior to and during the pandemic. While they found a small increase, logOR 0.33 (95% CI: 0.04 – 0.62), in the prevalence of loneliness compared to pre-pandemic assessments, there was significant heterogeneity. Importantly, only four of the included studies focused on the change in loneliness prevalence in older adults. Okely et al. [11] found an increase from 19 to 27% in 1000 community dwelling older adults who were part of the 1936 Lothian birth cohort (mean age of 84). Wong et al. [12] reported an increase from 40.5% to 70.1% in 593 older adults from an existing study of multimorbidity in primary care patients in Hong Kong (mean age 70.9). Herrera et al. [13] found an increase 43.0% to 47.8% in a random subsample of 720 older from a national survey of older adults in Chile (mean age 71.59) and Steptoe et al. [14] reported an increase from 30.4% to 33.7% in 4887 participants of the English Longitudinal Study of Ageing (mean age 70.4). Currently there are no data on the change in loneliness prevalence in Canada during the COVID-19, and in particular, whether the change in the prevalence of loneliness differed among sociodemographic and other health-related subgroups in order to identify high risk groups for potential interventions.

A growing body of research has identified risk and protective factors of loneliness during the COVID-19 pandemic in countries outside of Canada, ranging from small local studies to national studies, employing both cross-sectional and longitudinal data sets [15]. These include demographic, social, psychological, and health-related determinants; however, much of the evidence remains inconsistent, pointing to the need for additional research. For instance, being female was associated with higher rates of loneliness during COVID-19 in some studies [12, 16, 17], but not in others (e.g., [18, 19]. Also, advanced age was associated with greater loneliness [18, 20, 21], yet others have reported the opposite [16, 22], and a cluster of studies also reported higher levels loneliness among young and middle-aged adults compared to older adults [17, 23, 24]. Being unpartnered and living alone has been consistently identified as a risk factor associated with higher rates of loneliness during the pandemic [16, 18, 20, 21, 25, 26] but other aspects of the environment (housing type, rurality, etc.) have received little attention. Also absent is the inclusion of a full set of socio-economic status indicators (income, education, work status), although financial strain has been supported in one study [19]. Finally, indicators of poor health status have also been supported as risk factors for pandemic-related loneliness, including depression [27–30] anxiety [27, 29, 30], and chronic conditions [12, 21], whereas research on the effects of health behaviours (e.g., smoking, drinking, poor diet) on loneliness has been sparse.

The COVID-19 pandemic has led to many deleterious consequences for older persons worldwide. Given the clear evidence of the relationship between loneliness and health outcomes in older adults, [31] and its public health importance [32], it is imperative to better understand if, and how, loneliness has changed for older adults during the COVID-19 pandemic, and whom it has impacted most. In this paper we use population-based data from a well-characterized cohort, the Canadian Longitudinal Study on Aging (CLSA), to estimate the change in prevalence of loneliness among older adults prior to the onset of the COVID-19 pandemic and during the first year of the pandemic. Canada provides an interesting exemplar because while there is a universal healthcare system, health care delivery and public health responses to COVID-19 differed across the 10 Canadian provinces. Thus, we further examine a range of individual-level and population-level factors associated with reported loneliness during the COVID-19 pandemic to help to identify subgroups most at risk and provide direction for tailored interventions to address the impact of the COVID-19 pandemic.

## Methods

### Study design/setting

This study uses longitudinal data from the CLSA, a large, nationally generalizable sample of community-dwelling older adults aged 45–85 years residing in the ten provinces of Canada [33]. Sampling frames for recruitment into the CLSA included the Canadian Community Health Survey-Healthy Aging (a national population-based study conducted by Statistics Canada), provincial healthcare registration databases, and telephone sampling including random digit dialing [34]. Excluded from the cohort were full-time members of the armed forces, people living on First Nations reserves, residing in institutions, unable to respond in English or French, or with cognitive impairment. All participants provided informed consent, and the studies were approved by the Hamilton Integrated Research Ethics Board and by Institutions where each CLSA data collection site across Canada is situated.

### Participants

CLSA baseline data were collected on 51,338 participants in 2011–2015 and 48,893 participants (95% retention) were still enrolled at follow-up 1 (FUP1) in 2015–2018 and 44,817 (91.7%) provided data. Eligible CLSA participants (*n* = 42,511) were invited to take part in the CLSA COVID-19 study which was launched on April 15, 2020 and included a baseline questionnaire (Apr 15-May 29, 2020), weekly/biweekly/monthly symptoms questionnaires (Apr 23-Oct 16, 2020), and an exit questionnaire (Sep 29-Dec 29, 2020). CLSA FUP1 and the COVID-exit questionnaire both included a module on loneliness. A total of 28,559 (67.2%) CLSA participants completed the COVID-baseline questionnaire; and of those, 24,114 (84.4%) completed the COVID-exit questionnaire (Additional File 1a). Compared to those not participating (*n* = 18,343), those completing the Exit questionnaire (*n* = 24,114) were more likely to be aged 65–74 (34.7% vs. 33.1%), less likely to be < 55 (13.2% vs. 18.20%), and less likely to be a current smoker (5.8% vs. 8.9%). (Additional File 1b). These nominal and expected differences suggest that attrition is unlikely to have resulted in bias. This analysis uses data from FUP1 (2015–2018) to reflect the ‘Pre-COVID-19’ period and data from the COVID-exit (Sep 29 -Dec 29, 2020) to reflect the ‘During COVID-19’ period. A limited number of variables included from other timepoints are identified below in the Co-variates section.

### Loneliness scale

The 3-item UCLA loneliness scale is one of most widely used scales to assess loneliness and was adapted from its 20-item version for use with telephone surveys [35]. It has been extensively validated [36] and is the most commonly used measure in studies of loneliness and social isolation during the COVID-19 pandemic [37]. The 3 items are: 1) How often do you feel left out?; 2) How often do you feel isolated from others?; and 3) How often do you feel that you lack companionship? Items are scored with the response categories (1 = Hardly ever, 2 = Some of the time, 3 = Often) with an overall score ranging from 3 to 9. A dichotomous variable was created by classifying respondents in the top quintile of the distribution at FUP1 (≥ 5) as lonely and those in the bottom four quintiles (3–4) as not lonely, accounting for the skewed distribution of scores. This quintile-based cut-off approach has been used in other studies using the 3-item UCLA loneliness scale [37–40].

### Covariates

We conducted analyses to: 1) estimate the change in prevalence of loneliness among older adults pre-pandemic and during the pandemic; and 2) examine individual-level risk factors for loneliness during the pandemic (see statistical analysis section). Analyses were adjusted for (a) socio-demographic factors, (b) living conditions, (c) health status, and (d) health behaviours. Covariates were selected based on their association with loneliness as reported in the literature [15–30, 41–43]. These diverse risk factors were conceptualized using Socioecological (SE) theory, which posits that individual, social system, and environmental factors are interrelated and interdependent components of health [44, 45]. However, given the inconsistent findings in the extant COVID-19 literature, we do not posit explicit hypotheses.

#### Socio-demographic factors

Sociodemographic factors included: age (< 55, 55–64, 65–74, and ≥ 75), sex at birth (M/F), ethnicity (European or non-European background), education (less than secondary school graduation, secondary school graduation but no post-secondary education, post-secondary education degree or diploma) and total annual household income (< $20,000, $20,000-$49,999, $50,000-$99,999, $100,000-$149,999, and ≥ $150,000).

#### Living condition factors

Living conditions included: household composition (living alone, not living alone), dwelling type (house; apartment/condominium; and other types of dwelling including senior’s housing, mobile home, and hotel), living area (urban, rural), and province of residence (Alberta (AB), British Columbia (BC), Manitoba (MB), New Brunswick (NB), Newfoundland and Labrador (NL), Nova Scotia (NS), Ontario (ON), Prince Edward Island (PEI), Quebec (QC), and Saskatchewan (SK)).

#### Health status factors

Health status included depression and the number of chronic conditions experienced. Depression was operationalized using the 10-item Center for Epidemiologic Studies Short Depression Scale (CESD-10). A positive screen for depressive symptoms was based on a CESD-10 score of 10 or higher [46]. The number of chronic conditions was operationalized by summing the number of chronic conditions in 10 disease categories (musculoskeletal, respiratory, cardiovascular, endocrine-metabolic, neurological, gastrointestinal, genitourinary, ophthalmologic, renal, and cancer) and categorized into ‘none’, ‘one’, ‘two’, and ‘three or more’ chronic conditions.

#### Health behaviour factors

Health behaviour risk factors included: alcohol consumption (did not drink in the last 12 months, occasional, regular, or binge drinker), smoking status (never, former, or current smoker), physical activity, and social participation. Physical activity was assessed using the Physical Activity Scale for the Elderly (PASE) and dichotomized to meet the World Health Organization’s age specific guidelines for physical activity of at least 150 min of moderate-intensity or at least 75 min of vigorous-intensity physical activity per week [47, 48]. Social participation was assessed by summing the frequency of monthly participation in eight categories of community activities. Participants in the lowest sex- and age-specific quintiles were considered to have low social participation.

All previously mentioned covariates came from FUP1 except for sex, ethnicity, and education which were collected at CLSA baseline. The analyses examining individual-level risk factors of loneliness during the pandemic also included three early pandemic-related factors assessed at the COVID-19 baseline survey including: whether the participant was an essential worker, and the amount of time they spent alone weekday/weekend in the last month compared to the amount of time they usually spent alone (less than, the same, or more than usual).

### Statistical analysis

Descriptive statistics were calculated as mean (standard deviation (SD)) for continuous variables and frequency (%) for categorical variables at FUP1 and COVID-exit. Weighted generalized estimating equations (WGEE) were used to examine the change in prevalence of loneliness over time. WGEE was used to model longitudinal binary outcomes with monotonic missing data. The weights in WGEE are proportional to the reciprocal of the estimated probability that someone with the respondent’s covariate values and loneliness at FUP1 is still a respondent at COVID-exit. This helps to reduce bias in prevalence estimates at COVID-exit by accounting for factors associated with non-response to the COVID survey. Unadjusted and adjusted loneliness prevalence proportions and 95% confidence intervals were estimated for FUP1 and COVID-exit. The model was adjusted for socio-demographic (including time-dependent age), health status, social factors, living conditions, health behaviours, presence of depression, and province of residence, such that the adjusted population prevalence of loneliness reflects the change in prevalence if the distribution of all characteristics were the same at both time points. Adjusted estimates represent the predicted probability of loneliness at FUP1 and COVID-exit standardized with respect to age and assuming the same distribution for the socio-demographic, living conditions, health status, and health behaviour covariates. Prevalence estimates were calculated for the overall study population and subgroups based on sex, age, urban/rural status and province. Subgroup prevalence estimates at FUP1 and COVID-exit were calculated from models including an interaction term between the subgroup of interest and period (Pre-COVID-19 or During COVID-19). For subgroup analyses we focussed on adjusted estimates only to account for the different covariate distributions among the subgroups.

Lagged logistic regression was used to examine individual level factors associated with loneliness at COVID-exit, with loneliness at FUP1 considered as a predictor. A hierarchical set of models were estimated starting with loneliness at FUP1 as the only predictor and sequentially adding each set of covariates: sociodemographic factors, living conditions, health status, health behaviours, and pandemic-related factors. Because other cut-points have been used to define loneliness using the UCLA scale [37], we conducted sensitivity analyses using a cut-point of ≥ 4 and ≥ 6 to examine the robustness of our results. Although we were primarily interested in the prevalence of loneliness, we also ran lagged linear models using loneliness as a continuous variable and, because of its skewed distribution, ln (loneliness) to examine the robustness of our results when examining loneliness as a continuous variable. Because missing data were minimal (< 4% for all variables except income which was missing in 7%) complete case analysis was conducted. Multi-collinearity was assessed by estimating variance inflation factors (VIF); the maximum VIF was 3.12. All statistical analyses were conducted using SAS software version 9.4.

## Results

### Descriptive characteristics of CLSA participants pre-COVID-19 and during COVID-19

Cross-sectional descriptive characteristics of CLSA participants pre-COVID-19 (*n* = 44,817) and during COVID-19 (*n* = 24,114) are presented in Table 1. At the pre-pandemic timepoint, 51.19% of participants were women, 52.36% were aged 65 and older, 92.09% were of European background, and 65.94% had a household income of $50,000 or more. With respect to living characteristics, the majority lived in an urban area (85.07%), in a single-family dwelling (79.32%), and did not live alone (74.22%). Approximately two thirds of participants reported having two or more chronic conditions (65.32%). A similar proportion reported being a regular drinker (68.92%). While only 7.33% reported being a current smoker, 60.94% had smoked in the past. In comparing covariate distributions in the full pre-COVID-19 sample and the during COVID-19 sample, the differences in proportions were generally within two percentage points. There were differences of greater than two percentage points noted for social participation (a greater proportion of those who reported high social participation at the pre COVID-19 timepoint were respondents at the during COVID-19 timepoint) and urban/rural living status (a higher proportion of rural residents pre COVID-19 were respondents at the during COVID-19 timepoint).

**Table 1:** Descriptive characteristics of CLSA participants at Follow-Up 1 (2015–2018) and CLSA COVID-exit Questionnaire (Sep 29-Dec 29, 2020)

	**FUP1 (*n* = 44,817)**	**COVID-exit** **(*n* = 24,114)**
**VARIABLES**^a^	**n (%)**	**n (%)**
**SOCIO-DEMOGRAPHIC FACTORS**		
** Age Group**		
< 55	6598 (14.72)	890 (3.69)
55–64	14,751 (32.91)	7136 (29.59)
65–74	13,302 (29.68)	8856 (36.73)
75 +	10,166 (22.68)	7232 (29.99)
** Sex**		
Female	22,944 (51.19)	12,819 (53.16)
Male	21,873 (48.81)	11,295 (46.84)
** Ethnicity**		
Non-European	3132 (6.99)	1485 (6.16)
European	41,273 (92.09)	22,439 (93.05)
Missing	412 (0.92)	190 (0.79)
** Education**		
Less than secondary school graduation	2670 (5.96)	1109 (4.6)
Secondary school graduation, no post-secondary education	4735 (10.57)	2374 (9.84)
Post-secondary education/degree/diploma	37,309 (83.25)	20,588 (85.38)
Missing	103 (0.23)	43 (0.18)
** Annual Household Income**		
< $20,000	2083 (4.65)	861 (3.57)
$20,000-$49,999	9929 (22.15)	4855 (20.13)
$50,000-$99,999	15,124 (33.75)	8571 (35.54)
$100,000-$149,999	7810 (17.43)	4589 (19.03)
≥ $150,000	6616 (14.76)	3758 (15.58)
Missing	3255 (7.26)	1480 (6.14)
**LIVING CONDITIONS**		
** Number of People Living in the Same Household**		
Living alone	10,704 (23.88)	5991 (24.84)
Not	33,261 (74.22)	17,663 (73.25)
Missing	852 (1.9)	460 (1.91)
** Dwelling Type**		
House (single detached, semi-detached, duplex or townhouse)	35,549 (79.32)	18,740 (77.71)
Apartment or condominium	8006 (17.86)	4434 (18.39)
Other	1259 (2.81)	910 (3.77)
Missing	3 (0.01)	30 (0.12)
** Living Area**		
Rural	6660 (14.86)	4278 (17.74)
Urban	38,126 (85.07)	19,706 (81.72)
Missing	31 (0.07)	130 (0.54)
** Provinces/Territories**		
Newfoundland	2884 (6.44)	1370 (5.68)
Prince Edward Island	876 (1.95)	389 (1.61)
Nova Scotia	4010 (8.95)	2152 (8.92)
New Brunswick	1063 (2.37)	455 (1.89)
Quebec	8546 (19.07)	4353 (18.05)
Ontario	9831 (21.94)	5580 (23.14)
Manitoba	3978 (8.88)	2185 (9.06)
Saskatchewan	1063 (2.37)	568 (2.36)
Alberta	4490 (10.02)	2413 (10.01)
British Columbia	8073 (18.01)	4642 (19.25)
Missing^b^	3 (0)	7 (0.03)
**HEALTH STATUS**		
** Depression**		
Negative screen for depression	36,676 (81.84)	18,547 (76.91)
Positive screen for depression	6691 (14.93)	5219 (21.64)
Missing	1450 (3.24)	348 (1.44)
** Number of Chronic Conditions**		
0	5246 (11.71)	2794 (11.59)
1	8560 (19.1)	4775 (19.8)
2	8970 (20.01)	5064 (21)
3 +	20,308 (45.31)	10,532 (43.68)
Missing	1733 (3.87)	949 (3.94)
**HEALTH BEHAVIORS**		
** Type of Alcohol Drinker**		
Non-drinkers during last 12 months (including participants who never had alcohol)	6007 (13.4)	4350 (18.04)
Binge drinker	1996 (4.45)	1818 (7.54)
Regular drinker	30,889 (68.92)	15,004 (62.22)
Occasional drinker	5863 (13.08)	2756 (11.43)
Missing	62 (0.14)	186 (0.77)
** Type of Smoker**		
Current smoker	3285 (7.33)	1448 (6)
Former smoker	27,313 (60.94)	14,729 (61.08)
Never smoked	13,961 (31.15)	7523 (31.2)
Missing	258 (0.58)	414 (1.72)
** Physical Activity**^**c**^		
Low risk	12,988 (28.98)	7555 (31.33)
At risk	31,807 (70.97)	16,414 (68.07)
Missing	22 (0.05)	145 (0.6)
** Social Participation**^**d**^		
Low social participation	9243 (20.62)	4463 (18.51)
High social participation	34,513 (77.01)	19,379 (80.36)
Missing	1061 (2.37)	272 (1.13)

^a^Sex, ethnicity and education come from CLSA baseline for both FUP1 and COVID-exit; all other variables for FUP1 are from FUP1 sample; all other variables for COVID-exit are from the COVID-exit sample except for income, social participation, number of CCs, physical activity which come from FUP1 and number of people living in the same HH, dwelling type, living area (urban/rural), type of smoker, province which come from COVID baseline

^b^Three individuals who lived in Yukon and Nunavut at the time of the COVID-19 exit interview were added to the missing category and excluded from analyses

^c^Physical Activity: low risk = at least 150 min of moderate-intensity or at least 75 min of vigorous-intensity physical activity per week; high risk = less than 150 min of moderate-intensity or at least 75 min of vigorous-intensity physical activity per week

^d^Social Participation: low social participation = in the lowest sex- and age-specific quintiles of social participation; high social participation = in the top four sex- and age-specific quintiles of social participation

### Mean loneliness score and cross-sectional prevalence of loneliness pre-COVID-19 and during COVID-19 by participant characteristics

Table 2 presents the unadjusted mean loneliness scores and prevalence of loneliness by population subgroup at each timepoint. The overall mean loneliness score pre-COVID-19 was 3.86 (SD 1.33) and the overall mean loneliness score during COVID-19 was 4.29 (SD 1.56). Among subgroups, the mean loneliness scores pre-COVID-19 were highest for those with an annual income of less than $20,000 (4.86, SD 1.85), those who lived alone (4.46, SD 1.59), those who had low social participation (4.24, SD 1.62), those who did not live in a single-family dwelling or apartment (4.32, SD 1.61) and those who were current smokers (4.23, SD 1.62). The mean loneliness scores were higher for all subgroups during COVID-19, the only exception being those in the lowest income bracket (4.84, SD 1.83). The highest mean loneliness scores were reported for the following subgroups: those who lived alone (4.89, SD 1.76), those whose income was less than $20,000 (4.84, SD 1.83) and $20,000 or more but less than $50,000 (4.53, SD 1.68), current smokers (4.51, SD 1.72), and those who lived in an apartment or condominium (4.59, SD 1.69) or other type of non-house dwelling (4.50, SD 1.73).

**Table 2:** Mean loneliness score and prevalence of loneliness pre-COVID-19 (2015–2018) and during COVID-19 (Sep 29-Dec 29, 2020)

	**Pre-COVID-19 (*n* = 44,817)**				**During COVID-19 (*n* = 24,114)**			
	**LONELINESS SCORE** **(*n* = 44,374)**		**Lonely (*n* = 10,285)**		**LONELINESS SCORE (*n* = 23,619)**		**Lonely (*n* = 8,587)**	
**VARIABLES**^a^	**Mean**	**Std**	**n**	**%**	**Mean**	**Std**	**n**	**%**
**OVERALL**	**3.86**	**1.33**			**4.29**	**1.56**		
**Age Group**								
< 55	3.86	1.35	1513	**22.93**	4.32	1.57	330	**37.08**
55–64	3.88	1.37	3440	**23.32**	4.30	1.57	2579	**36.14**
65–74	3.81	1.29	2852	**21.44**	4.31	1.58	3186	**35.98**
75 +	3.90	1.31	2480	**24.40**	4.26	1.52	2492	**34.46**
**Sex**								
Female	3.95	1.38	5793	**25.25**	4.46	1.63	5201	**40.57**
Male	3.77	1.27	4492	**20.54**	4.09	1.44	3386	**29.98**
**Ethnicity**								
Non-European	4.05	1.47	887	**28.32**	4.35	1.64	541	**36.43**
European	3.84	1.32	9282	**22.49**	4.29	1.55	7975	**35.54**
Missing	4.07	1.49	116	**28.16**	4.26	1.64	71	**37.37**
**Education**								
Less than secondary school graduation	4.17	1.53	839	**31.42**	4.29	1.62	377	**33.99**
Secondary school graduation, no post-secondary education	3.88	1.34	1120	**23.65**	4.28	1.58	834	**35.13**
Post-secondary education/degree/diploma	3.84	1.31	8294	**22.23**	4.29	1.55	7360	**35.75**
Missing	4.29	1.65	32	**31.07**	4.53	1.94	16	**37.21**
**Annual Household Income**								
< $20,000	4.86	1.85	984	**47.24**	4.84	1.83	411	**47.74**
$20,000-$49,999	4.14	1.47	3114	**31.36**	4.53	1.68	2001	**41.22**
$50,000-$99,999	3.79	1.26	3220	**21.29**	4.27	1.54	3007	**35.08**
$100,000-$149,999	3.60	1.08	1261	**16.15**	4.14	1.45	1469	**32.01**
≥ $150,000	3.49	0.98	837	**12.65**	4.04	1.38	1108	**29.48**
Missing	4.08	1.49	869	**26.70**	4.44	1.65	591	**39.93**
**Number of People Living in the Same Household**								
Living alone	4.46	1.59	4150	**38.77**	4.89	1.76	3043	**50.79**
Not	3.67	1.17	5969	**17.95**	4.08	1.42	5352	**30.30**
Missing	4.06	1.48	166	**19.48**	4.53	1.64	192	**41.74**
**Dwelling Type**								
House (single detached, semi-detached, duplex or townhouse)	3.77	1.26	7319	**20.59**	4.21	1.51	6326	**33.76**
Apartment or condominium	4.18	1.53	2519	**31.46**	4.59	1.69	1905	**42.96**
Other	4.32	1.61	446	**35.42**	4.50	1.73	347	**38.13**
Missing	4.00	1.00	1	**33.33**	4.04	1.40	9	**30.00**
**Living Area**								
Rural	3.84	1.32	1489	**22.36**	4.13	1.47	1346	**31.46**
Urban	3.87	1.33	8788	**23.05**	4.32	1.57	7193	**36.50**
Missing	4.10	1.54	8	**25.81**	4.48	1.85	48	**36.92**
**Provinces/Territories**								
Newfoundland	3.78	1.25	613	**21.26**	4.01	1.41	380	**27.74**
Prince Edward Island	3.83	1.34	187	**21.35**	4.01	1.41	116	**29.82**
Nova Scotia	3.75	1.30	788	**19.65**	4.05	1.49	624	**29.00**
New Brunswick	3.99	1.45	287	**27.00**	4.11	1.58	136	**29.89**
Quebec	3.98	1.34	2251	**26.34**	4.31	1.55	1576	**36.20**
Ontario	3.83	1.32	2136	**21.73**	4.34	1.58	2053	**36.79**
Manitoba	3.83	1.33	900	**22.62**	4.45	1.63	866	**39.63**
Saskatchewan	4.06	1.45	304	**28.60**	4.42	1.58	237	**41.73**
Alberta	3.86	1.35	1068	**23.79**	4.36	1.55	917	**38.00**
British Columbia	3.83	1.33	1749	**21.66**	4.31	1.56	1677	**36.13**
Missing^b^	5	1	2	66.67	5.57	1.99	5	**71.43**
**Depression**								
Negative screen for depression	3.64	1.08	6244	**17.02**	3.90	1.22	4781	**25.78**
Positive screen for depression	5.08	1.82	3730	**55.75**	5.70	1.80	3746	**71.78**
Missing	3.95	1.42	311	**21.45**	4.39	1.64	60	**17.24**
**Number of Chronic Conditions**								
0	3.62	1.11	858	**16.36**	4.04	1.39	826	**29.56**
1	3.68	1.18	1508	**17.62**	4.09	1.41	1468	**30.74**
2	3.77	1.23	1890	**21.07**	4.23	1.53	1725	**34.06**
3 +	4.04	1.45	5612	**27.63**	4.47	1.65	4221	**40.08**
Missing	3.89	1.40	417	**24.06**	4.34	1.62	347	**36.56**
**Type of Alcohol Drinker**								
Non-drinkers during last 12 months (including participants who never had alcohol)	4.11	1.57	1757	**29.25**	4.36	1.63	1603	**36.85**
Binge drinker	3.90	1.40	459	**23.00**	4.29	1.56	648	**35.64**
Regular drinker	3.76	1.23	6328	**20.49**	4.24	1.51	5207	**34.70**
Occasional drinker	4.11	1.49	1725	**29.42**	4.45	1.66	1109	**40.24**
Missing	4.13	1.78	16	**25.81**	4.15	1.44	20	**10.75**
**Type of Smoker**								
Current smoker	4.23	1.62	1019	**31.02**	4.51	1.72	577	**39.85**
Former smoker	3.84	1.30	6146	**22.50**	4.29	1.56	5256	**35.68**
Never smoked	3.82	1.30	3059	**21.91**	4.24	1.52	2599	**34.55**
Missing	3.83	1.22	61	**23.64**	4.38	1.57	155	**37.44**
**Physical Activity**^**c**^								
Low risk	3.68	1.17	2358	**18.16**	4.18	1.46	2501	**33.10**
At risk	3.93	1.39	7921	**24.90**	4.34	1.60	6036	**36.77**
Missing	4.31	2.06	6	**27.27**	4.36	1.67	50	**34.48**
**Social Participation**^**d**^								
Low social participation	4.24	1.62	3037	**32.86**	4.51	1.73	1783	**39.95**
High social participation	3.75	1.22	7012	**20.32**	4.24	1.51	6705	**34.60**
Missing	4.10	1.52	236	**22.24**	4.42	1.67	99	**36.40**

^a^Sex, ethnicity and education come from CLSA baseline for both FUP1 and COVID-exit; all other variables for FUP1 are from FUP1 sample; all other variables for COVID-exit are from the COVID-exit sample except for income, social participation, number of CCs, physical activity which come from FUP1 and number of people living in the same HH, dwelling type, living area (urban/rural), type of smoker, province which come from COVID baseline

^b^Three individuals who lived in Yukon and Nunavut at the time of the COVID-19 exit interview were added to the missing category and excluded from analyses

^c^Physical Activity: low risk = at least 150 min of moderate-intensity or at least 75 min of vigorous-intensity physical activity per week; high risk = less than 150 min of moderate-intensity or at least 75 min of vigorous-intensity physical activity per week

^d^Social Participation: low social participation = in the lowest sex- and age-specific quintiles of social participation; high social participation = in the top four sex- and age-specific quintiles of social participation

Similar patterns were seen when we examined the prevalence of loneliness by subgroups. Subgroups with a prevalence higher than 30% pre-COVID-19 were: education less than secondary school graduation (31.42%), income < $20,000 (47.24%) and $20,000 or more but less than $50,000 (31.36%), living alone (38.77%), living in an apartment/condo (31.46%) or other type of non-house dwelling (35.42%), being a current smoker (31.02%), and low social participation (32.86%). These subgroups also generally had the highest prevalence of loneliness during COVID-19. In addition to these subgroups, those with a prevalence higher than 40% during COVID-19 were: women (40.57%), those with 3 + chronic conditions (40.08%), occasional drinkers (40.24%), and residing in Saskatchewan (41.73%).

A steep inverse pattern was noted with respect to loneliness according to income bracket. Those in the highest income category of ≥ $150,000 had the lowest prevalence of loneliness (12.65%) whereas those in the lowest income bracket of < $20,000 had the highest prevalence of loneliness (47.24%) pre-COVID-19. A positive association was noted for number of chronic conditions. Of those with no chronic conditions, 16.36% reported being lonely, and this proportion increased to 27.63% among those with 3 or more chronic conditions. These patterns were retained during COVID-19, though the prevalence was higher and the increases less steep. Interestingly, a consistent pattern was not noted for age group. The highest prevalence of loneliness by age pre-COVID-19 was reported among those who were 75 and older and the lowest prevalence of loneliness was reported among those who were aged 65–74. During COVID-19, however, the prevalence of loneliness was inversely associated with age group.

### Change in the prevalence of loneliness from pre-COVID-19 to during COVID-19

**Fig. 1:**
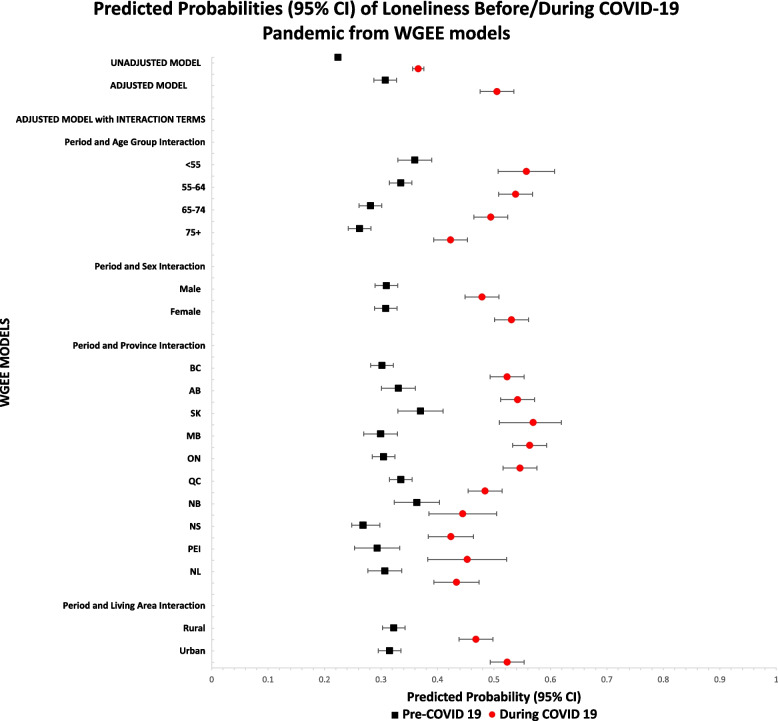
Pre-pandemic and during pandemic prevalence of loneliness among population-based subgroups of Canadian Longitudinal Study on Aging (CLSA) participants. Adjusted models include socio-demographic factors (including time-dependent age), health status, social factors, living conditions, health behaviours, presence of depression, and province of residence. BC (British Columbia); AB (Alberta); SK (Saskatchewan); MB (Manitoba); ON (Ontario); QC (Quebec); NB (New Brunswick); NS (Nova Scotia); PEI (Prince Edward Island); NL (Newfoundland and Labrador)

Figure 1 displays the results from the WGEE analyses used to examine the change in prevalence of loneliness from pre-COVID-19 to during COVID-19. The unadjusted prevalence of loneliness pre-pandemic was 22.37% (CI: 21.95%-22.79%); the prevalence increased to 36.59% (CI: 35.96%-37.23%) during COVID-19. After standardizing with respect to age and adjusting for socio-demographic, living conditions, health status, and health behaviour covariates the predicted probability of loneliness was 30.75% (CI: 28.72%-32.85%) pre-COVID-19 and 50.53% (CI: 48.01%-53.06%) during COVID-19. This reflected an absolute increase in loneliness from pre-COVID-19 to during COVID-19 of 14.23% (unadjusted) and 19.78% (adjusted) and a relative increase of 63.61% (unadjusted) and 64.33% (adjusted). These data indicate that pre-pandemic to pandemic loneliness increased and increased further after standardizing for age and adjusting for other covariates. After adjusting for all other covariates loneliness decreased with age both pre-COVID-19 and during COVID-19; the oldest age group also had the lowest absolute increase in loneliness during the pandemic. While women and those living in urban settings had a slightly lower predicted probability of loneliness pre-pandemic, the absolute change in loneliness during the pandemic for these groups was larger compared to their counterparts (women 22.26% vs. men 16.95% and 20.84% urban vs. 14.55% rural). The pre-pandemic predicted probability of loneliness varied considerably among provinces from 26.81% in Nova Scotia to 37.00% in Saskatchewan; the greatest absolute change from pre-pandemic to during the pandemic was seen for Manitoba (26.40%) and Ontario (24.16%).

### Predictors of loneliness during the COVID-19 pandemic

The lagged logistic analyses examined factors associated with loneliness during COVID-19 (Table 3). Pre-pandemic loneliness was a statistically significant predictor of loneliness during the pandemic period, increasing the odds of loneliness during the pandemic almost five-fold (aOR 4.87; CI 4.49–5.28). Predictors of loneliness during the pandemic period included the socio-demographic characteristics of age (increased odds of loneliness in younger age groups; (aOR 1.41; CI 1.23–1.63) for < 55 vs 75 + years), sex (aOR 1.34; CI 1.25–1.43 for women vs. men), and education (aOR 0.73; CI 0.61–0.86 for those graduating secondary school but no post-secondary education vs. those with post-secondary education), but not income. Living conditions associated with loneliness during the pandemic period included living alone (aOR 1.39; CI 1.27–1.52) and living in an urban area (aOR 1.18; CI 1.07–1.30). With respect to province of residence, increased odds of loneliness were reported in all provinces relative to NS, except the other Atlantic provinces (PEI, NB, NL). Health-related factors associated with loneliness included depression (aOR 2.08; CI 1.88–2.30) and having two, or three or more chronic conditions (aOR 1.16; CI 1.03–1.31 and aOR 1.34; CI 1.20–1.50 respectively), relative to no chronic conditions. Being a regular drinker (aOR 1.15; CI 1.04–1.28) relative to not drinking in the past 12 months was the only health behaviour independently associated with loneliness. Of the pandemic-related factors, essential workers had lower odds of loneliness compared to non-essential workers (aOR 0.77; CI 0.69–0.87). Interestingly, those reporting spending less time alone than usual on weekdays and on weekends had higher odds of loneliness compared to those reporting spending the same amount of time alone (aOR 1.32; CI 1.19–1.46 and aOR 1.27; CI 1.14–1.41 respectively). Similar results in terms of the direction and confidence interval coverage were found in the sensitivity analyses using different cut-points to define loneliness (Additional File 2a and b) and loneliness as a continuous variable (Additional File 2c and d).

**Table 3:** Predictors of loneliness during the COVID-19 pandemic, adjusted for loneliness and participant characteristics in the pre-pandemic period

Variables^a^	Model 1 (*n* = 23,544) OR (95% CI)	Model 2 (*n* = 22,058) OR (95% CI)	Model 3 (*n* = 22,018) OR (95% CI)	Model 4 (*n* = 21,176) OR (95% CI)	Model 5 (*n* = 20,975) OR (95% CI)	Model 6 (*n* = 20,033) OR (95% CI)
**Loneliness at FUP1 (ref = "not lonely")**						
Lonely	**5.93 (5.54,6.35)**	**5.83 (5.42,6.28)**	**5.59 (5.19,6.03)**	**4.79 (4.43,5.18)**	**4.8 (4.44,5.2)**	**4.87 (4.49,5.28)**
**Age (ref = "75 + ")**						
< 55	-	1.08 (0.97,1.21)	**1.19 (1.06,1.34)**	**1.33 (1.17,1.51)**	**1.35 (1.19,1.53)**	**1.41 (1.23,1.63)**
55–64	-	1.09 (1,1.19)	**1.18 (1.08,1.29)**	**1.29 (1.17,1.42)**	**1.3 (1.18,1.44)**	**1.35 (1.21,1.49)**
65–74	-	0.99 (0.91,1.09)	1.06 (0.97,1.16)	**1.12 (1.03,1.23)**	**1.14 (1.03,1.25)**	**1.16 (1.05,1.27)**
**Sex (ref = "Male")**						
Female	-	**1.52 (1.43,1.62)**	**1.46 (1.37,1.55)**	**1.37 (1.29,1.46)**	**1.39 (1.3,1.48)**	**1.34 (1.25,1.43)**
**Ethnicity (ref = "European")**						
Non-European	-	0.97 (0.85,1.09)	0.97 (0.86,1.1)	0.94 (0.83,1.07)	0.97 (0.85,1.1)	0.94 (0.83,1.08)
**Education (ref = "Post-secondary education/degree/diploma")**						
Secondary school graduation, no post-secondary education	-	0.69 (0.59,0.81)	**0.73 (0.62,0.85)**	**0.7 (0.6,0.83)**	**0.72 (0.61,0.85)**	**0.73 (0.61,0.86)**
Less than secondary school graduation	-	0.88 (0.79,0.97)	**0.9 (0.81,1)**	**0.89 (0.8,0.99)**	**0.89 (0.8,0.99)**	0.9 (0.81,1.01)
**HH Income (ref = " ≥ $150,000")**						
< $20,000	-	**1.36 (1.14,1.62)**	1.09 (0.9,1.31)	0.93 (0.76,1.13)	0.94 (0.77,1.15)	0.98 (0.8,1.2)
$20,000-$49,999	-	**1.29 (1.16,1.43)**	**1.17 (1.04,1.31)**	1.06 (0.95,1.19)	1.08 (0.96,1.21)	1.06 (0.94,1.2)
$50,000-$99,999	-	**1.16 (1.06,1.28)**	**1.14 (1.04,1.25)**	1.08 (0.98,1.19)	1.08 (0.98,1.19)	1.07 (0.97,1.19)
$100,000-$149,999	-	1.1 (0.99,1.21)	1.09 (0.99,1.21)	1.07 (0.96,1.18)	1.07 (0.96,1.18)	1.08 (0.97,1.2)
≥ $150,000						
**Number of people living in the same HH (ref = "Not living alone”)**						
Living alone	-	-	**1.5 (1.38,1.62)**	**1.52 (1.4,1.65)**	**1.51 (1.39,1.65)**	1.39 (1.27,1.52)
**Dwelling Type (ref = "House (single detached, semi-detached, duplex or townhouse)")**						
Apartment or condominium	-	-	1.04 (0.96,1.14)	1.02 (0.93,1.11)	1.03 (0.94,1.12)	1.01 (0.92,1.11)
Other	-	-	1.02 (0.82,1.27)	0.97 (0.78,1.22)	0.97 (0.78,1.22)	0.98 (0.77,1.24)
**Living Area (ref = "Rural")**						
Urban	-	-	**1.21 (1.1,1.32)**	**1.2 (1.09,1.32)**	**1.2 (1.09,1.32)**	**1.18 (1.07,1.3)**
**Province**^b^** (ref = "NS")**						
Alberta	-	-	**1.46 (1.27,1.68)**	**1.49 (1.28,1.73)**	**1.5 (1.29,1.75)**	**1.55 (1.32,1.81)**
British Columbia	-	-	**1.33 (1.18,1.51)**	**1.39 (1.21,1.59)**	**1.41 (1.23,1.62)**	**1.43 (1.24,1.64)**
Manitoba	-	-	**1.62 (1.41,1.88)**	**1.72 (1.48,2.01)**	**1.75 (1.49,2.04)**	**1.72 (1.46,2.02)**
New Brunswick	-	-	0.96 (0.75,1.24)	1 (0.77,1.31)	1.03 (0.79,1.34)	1.02 (0.77,1.33)
Newfoundland and Labrador	-	-	0.9 (0.76,1.07)	0.93 (0.78,1.11)	0.95 (0.79,1.13)	0.94 (0.78,1.13)
Ontario	-	-	**1.48 (1.31,1.67)**	**1.51 (1.33,1.73)**	**1.54 (1.35,1.76)**	**1.54 (1.34,1.76)**
Prince Edward Island	-	-	1.16 (0.89,1.51)	1.2 (0.91,1.58)	1.26 (0.95,1.66)	1.3 (0.97,1.73)
Québec	-	-	**1.22 (1.08,1.39)**	**1.29 (1.12,1.47)**	**1.29 (1.12,1.48)**	**1.3 (1.13,1.5)**
Saskatchewan	-	-	**1.52 (1.22,1.9)**	**1.62 (1.29,2.04)**	**1.67 (1.33,2.1)**	**1.76 (1.39,2.23)**
**Depression (ref = "Negative screen for depression")**						
Positive screen for depression	-	-	-	**2.11 (1.91,2.31)**	**2.12 (1.92,2.33)**	**2.08 (1.88,2.3)**
**Number of CCs (ref = "0")**						
1	-	-	-	1.06 (0.95,1.19)	1.06 (0.94,1.19)	1.06 (0.94,1.19)
2	-	-	-	**1.16 (1.04,1.3)**	**1.16 (1.04,1.3)**	**1.16 (1.03,1.31)**
3 +	-	-	-	**1.35 (1.21,1.51)**	**1.35 (1.21,1.51)**	**1.34 (1.2,1.5)**
**Type of Alcohol Drinker (ref = "Did not drink in the last 12 months")**						
Binge drinker	-	-	-	-	1.06 (0.88,1.27)	0.99 (0.82,1.2)
Regular drinker	-	-	-	-	**1.18 (1.06,1.31)**	**1.15 (1.04,1.28)**
Occasional drinker	-	-	-	-	1.11 (0.98,1.26)	1.11 (0.97,1.27)
**Type of Smoker (ref = "Never smoked")**						
Current smoker	-	-	-	-	1.1 (0.95,1.27)	1.13 (0.98,1.31)
Former smoker	-	-	-	-	1.07 (1,1.14)	1.06 (0.99,1.14)
**Physical Activity**^**c**^ **(ref = "Low risk")**						
At risk	-	-	-	-	1.03 (0.96,1.1)	1.03 (0.96,1.11)
**Social Participation**^**d**^ **(ref = "High social participation")**						
Low social participation	-	-	-	-	0.97 (0.9,1.06)	0.99 (0.91,1.08)
**CHANGE in average weekday alone time during the day (ref = "Same")**						
Less than usual	-	-	-	-	-	**1.32 (1.19,1.46)**
More than usual	-	-	-	-	-	1.1 (0.99,1.22)
**CHANGE in average weekend alone time during the day (ref = "Same")**						
Less than usual	-	-	-	-	-	**1.27 (1.14,1.41)**
More than usual	-	-	-	-	-	0.97 (0.87,1.08)
**Essential Worker (ref = "Not works outside of residence")**						
Yes	-	-	-	-	-	**0.77 (0.69,0.87)**
No	-	-	-	-	-	0.99 (0.9,1.1)

^a^Sex, ethnicity and education come from CLSA baseline; all other variables come from FUP1 sample except for change in average weekday alone time during the day, change in average weekend alone time during the day, essential worker which come from COVID baseline sample

^b^Three individuals who lived in Yukon and Nunavut at the time of the COVID-19 exit interview were added to the missing category and excluded from analyses

^c^Physical Activity: low risk = at least 150 min of moderate-intensity or at least 75 min of vigorous-intensity physical activity per week; high risk = less than 150 min of moderate-intensity or at least 75 min of vigorous-intensity physical activity per week

^d^Social Participation: low social participation = in the lowest sex- and age-specific quintiles of social participation; high social participation = in the top four sex- and age-specific quintiles of social participation

## Discussion

Our results show that the prevalence of loneliness among community dwelling older adults increased from 22.4% pre-pandemic to 36.5% during the COVID-19 pandemic; this increased to over 50% after standardizing with respect to age and adjusting for socio-demographic, living conditions, health status, and health behaviours. As has been repeatedly noted for various other outcomes, the pandemic did not impact all subgroups of the population in the same way with respect to loneliness. Not only was a higher prevalence of loneliness seen for women than men during the pandemic, the change in prevalence from the pre-pandemic period to the end of the first calendar year of the pandemic was also greater for women. Interestingly, age was inversely associated with loneliness during the pandemic. This may be because the relative impact of public health restrictions was greater on the younger age groups, who had more social engagement and were less lonely to begin with. The differences in prevalence of loneliness by province likely reflect differences in public health measures in place, as well as differences in the number and rate of COVID-19 cases among the provinces. For example, the Atlantic provinces experienced much lower rates of COVID-19 during the first wave, whereas BC, ON and QC experienced the highest rates of COVID-19. There were also differential policy responses as a result of public health guidance and political/economic tensions.

Our prevalence estimates are consistent with many other studies [10, 49]. However, there are some differences likely due to the measurement tools used, timeframes of observation, and contexts. The only other Canadian data were reported by Savage et al. [50] who conducted an online survey of 4,879 retired teachers in Ontario in May 2020. They found that 43.1% self-reported being lonely at least some of the time in the past 7 days. Several longitudinal studies have also reported data on loneliness collected at multiple points during the pandemic. Kotwal et al. [27] collected data from April to June 2020 from 151 older adults recruited from a geriatric outpatient clinic and two senior centres in California. They reported levels of severe loneliness varied from 23 to 36% but did not change in a consistent way over time. It should be noted that while they used the UCLA loneliness scale, they used a higher cut-point (equivalent to ≥ 6 on our scale). In a national sample of 2,337 US adults aged 50 years or older collected in May 2020, Choi et al. [16] found 26.8% reported being lonely for at least 1 day in the previous week. They also found that the prevalence did not change over subsequent waves. Other authors focussed on the longitudinal change in continuous loneliness scores. Luchetti et al. [51] collected data from a nationwide sample of 1,545 adults 18 years and older between Feb to April 2020 using the 11 item UCLA Loneliness Scale. They found that older adults reported lower loneliness on average compared to younger age groups overall but had an increase in loneliness during the acute phase of the pandemic. Losada-Baltar et al. [17] collected loneliness data from 1,549 adults 18 years and older from Spain using a single item with a 10-point Likert scale and found a linear increase in loneliness scores over time (March to May 2020) for both older adults and younger age groups. Importantly, we could find no other studies that reported on the change in prevalence of loneliness from the pre-pandemic period to the pandemic period.

Our lagged logistic regression results show that being lonely prior to the pandemic increased the odds of being lonely during the pandemic by almost five-fold. The sociodemographic characteristics of age, sex, and education were significant predictors of pandemic loneliness, and this finding aligns with other research [52]. Interestingly, the odds of loneliness decreased with age. Similar findings were reported by Allen et al. [23] in a large national cross-sectional sample of adults aged ≥ 18 years. However, we found that ethnicity and household income did not remain statistically significant in the fully adjusted model. Being female, in a younger age bracket, and in a higher education group increased the odds of loneliness during the pandemic. The pattern seen for education was not expected, but may be related to associated differences in occupation, such as those in higher education groups holding positions that allowed working from home during the pandemic. Alternatively, it could be that those in higher education brackets held employment positions that required greater isolation from family and friends. The model was also adjusted for being an essential worker during the pandemic, which was shown to be protective against pandemic loneliness.

Of the factors related to living conditions, living alone, living in an urban area, and living in a province outside of the Atlantic region were statistically significant predictors of loneliness during the pandemic. Again, these findings likely reflect the differences across the country in the pandemic itself, such as the number of cases locally and the associated public health measures in place. While there were strict quarantine requirements for entry into the Atlantic region during the period of the pandemic under investigation, there were also fewer lockdowns and less severe restrictions on social interactions.

Health status, as measured by number of chronic conditions and a positive screen for depressive symptoms prior to the pandemic predicted loneliness during the pandemic. Multiple studies have reported that perceived loneliness during the pandemic was associated with depression [27–30] and anxiety [27, 29, 30]. An earlier study using CLSA data that focussed on the outcome of depression found a similar relationship between pre-pandemic loneliness and depressive symptoms [53], suggesting a possible bidirectional association. Further longitudinal investigation of this association is warranted.

We also found that engaging in the behaviours of drinking alcohol, smoking cigarettes, and low social participation pre-pandemic were significant predictors of pandemic loneliness, but low physical activity was not. These relationships were not unexpected as these factors have been previously shown to be associated with loneliness prior to the pandemic [52].

### Strengths and limitations

Our national population-based study examines longitudinal changes in loneliness from the pre-pandemic to the end of the first year of the pandemic in Canada. Our sample estimates of prevalence are unique in that they take into account that the pandemic sample was a subset of the pre-pandemic sample. While we cannot eliminate the possibility of response bias, our statistical methods helped to reduce its impact and estimate loneliness as if all sociodemographic and health variables were the same at both timepoints. The large sample size of the CLSA allowed for analyses that highlight important differences among population subgroups and demonstrated that not all subgroups of the population may experience the impacts of the pandemic equally. However, a limitation of the CLSA is its largely white population, whereas we know that the COVID-19 pandemic has differentially affected ethnic and visible minority populations to a greater extent [54]. An added strength of the study is the use of the 3-item UCLA loneliness scale, the loneliness measure most commonly used in studies of epidemics or pandemics [37]. Studies using this scale have, however, used a number of different cut points, making comparisons to other findings challenging. The cut-point we used (≥ 5) may have resulted in a conservative estimate of loneliness. In addition, because we classified loneliness as a dichotomous variable, we may not have been able to fully capture change in loneliness. For example, if individuals scored above the cut point for loneliness at FUP1, even if their loneliness score increased during the pandemic period, no change would be seen in their classification of loneliness. However, our sensitivity analyses using different cut-points to define loneliness and using loneliness as a continuous variable supported our primary findings. Finally, while our data capture loneliness during a critical phase of the pandemic, we cannot say whether loneliness persisted, dissipated, or increased during subsequent waves. Having population-based data in an existing cohort is necessary to fully understand the long-term impact of these patterns.

## Conclusion

Many recommendations for public health action to combat social isolation and loneliness pre-dated COVID-19, but the pandemic has heightened this call for action [55]. New frameworks, such as the Systematic Framework of Cross-Sector Integration and Action Across the Life Span (SOCIAL)[32] have been proposed to more holistically guide public health and can be used to identify evidence gaps. Our findings suggest that loneliness during the pandemic was associated with pre-pandemic loneliness and individual level sociodemographic factors, living and working conditions, health status, and health behaviours. Thus, public health measures aimed at reducing loneliness during a pandemic must incorporate multifactor interventions fostering positive health behaviours and consider targeting those at high risk for loneliness. These could follow a multi-level approach based on an educate, assess and respond (EAR) framework that incorporates healthy lifestyles, surveillance, and tailored approaches that was recently proposed by Holt-Lunstad and Perissinotto [56].

## Supplementary Information

**Additional file 1.** a: Comparison of sociodemographic and health characteristics of the CLSA Baseline cohort and participants completing the COVID-19 Exit Questionnaire. b: Comparison of sociodemographic and health characteristics from CLSA Follow-up1 for participants who completed the COVID-19 Exit questionnaire (*n*=24,114) and participants who did not completethe COVID-19 Exit questionnaire (*n*=18,343).

**Additional file 2.** a: Predictors of loneliness during the COVID-19 pandemic, adjusted for pre-pandemic loneliness and participant characteristics in the pre-pandemic period using an alternative cut-off value “≥4”. b: Predictors of loneliness during the COVID-19 pandemic, adjusted for pre-pandemic loneliness and participant characteristics in the pre-pandemic period using an alternative cut-off value “≥6”. c: Predictors of loneliness during the COVID-19 pandemic, adjusted for pre-pandemic loneliness and participant characteristics in the pre-pandemic period using lagged linear regression. d: Predictors of ln(loneliness) during the COVID-19 pandemic, adjusted for pre-pandemic ln(loneliness) and participant characteristics in the pre-pandemic period using lagged linear regression.

## Data Availability

Data are available from the Canadian Longitudinal Study on Aging (https://www.clsa-elcv.ca) for researchers who meet the criteria for access to de-identified CLSA data. Custom code that supports the results of this study can be made available upon request from the corresponding author.

## Abbreviations

ADL: Activities of Daily Living
CLSA: Canadian Longitudinal Study on Aging
CESD-10: Center for Epidemiologic Studies Short Depression Scale
ELSA: English Longitudinal Study on Ageing
FUP1: Follow-up 1
PASE: Physical Activity Scale for the Elderly
WGEE: Weighted generalized estimating equations
