# Supplementary material for: Increased prevalence of loneliness and associated risk factors during the COVID-19 pandemic: findings from the Canadian Longitudinal Study on Aging (CLSA)
Source: BMC Public Health. 2023 May 12;23:872. doi: 10.1186/s12889-023-15807-4 (PMC10175060; doi:10.1186/s12889-023-15807-4)
Supplement: Supplementary file 1 — Additional file 1. a: Comparison of sociodemographic and health characteristics of the CLSA Baseline cohort and participants completing the COVID-19 Exit Questionnaire. b: Comparison of sociodemographic and health characteristics from CLSA Follow-up1 for participants who completed the COVID-19 Exit questionnaire (n=24,114) and participants who did not completethe COVID-19 Exit questionnaire (n=18,343). [file 12889_2023_15807_MOESM1_ESM.docx]

Additional File 1a. Comparison of sociodemographic and health characteristics of the CLSA Baseline cohort and participants completing the COVID-19 Exit Questionnaire.

|  | **BASELINE**  **(n=51,338)** | | **CLSA Follow-up 1 (n=44,817)** | | **Completed**  **COVID-19 EXIT (n=24,114)** | |
| --- | --- | --- | --- | --- | --- | --- |
|  | **n** | **%** | **n** | **%** | **n** | **%** |
| **Age Group^1^** |  |  |  |  |  |  |
| <55 | 13427 | 26.15 | 6598 | 14.72 | 890 | 3.69 |
| 55-64 | 16420 | 31.98 | 14751 | 32.91 | 7136 | 29.59 |
| 65-74 | 11996 | 23.37 | 13302 | 29.68 | 8856 | 36.73 |
| 75+ | 9495 | 18.5 | 10166 | 22.68 | 7232 | 29.99 |
| **Sex** |  |  |  |  |  |  |
| Female | 26155 | 50.95 | 22944 | 51.19 | 12819 | 53.16 |
| Male | 25183 | 49.05 | 21873 | 48.81 | 11295 | 46.84 |
| **Ethnicity** |  |  |  |  |  |  |
| European | 47105 | 92.68 | 41273 | 92.95 | 22439 | 93.79 |
| Non-European | 3718 | 7.32 | 3132 | 7.05 | 1485 | 6.21 |
| **Annual Household Income** |  |  |  |  |  |  |
| Less than $20,000 | 2913 | 6.07 | 2083 | 5.01 | 861 | 3.80 |
| $20,000-$49,999 | 12209 | 25.42 | 9929 | 23.89 | 4855 | 21.45 |
| $50,000-$99,999 | 17127 | 35.66 | 15124 | 36.39 | 8571 | 37.87 |
| $100,000-$149,999 | 8739 | 18.20 | 7810 | 18.79 | 4589 | 20.27 |
| $150,000 or more | 7039 | 14.66 | 6616 | 15.92 | 3758 | 16.60 |
| **Number of People Living in the Same Household** |  |  |  |  |  |  |
| Living alone | 11747 | 22.89 | 10704 | 24.35 | 5991 | 25.33 |
| Not living alone | 39564 | 77.11 | 33261 | 75.65 | 17663 | 74.67 |
| **Living Area** |  |  |  |  |  |  |
| Rural | 9634 | 18.77 | 6660 | 14.87 | 4278 | 17.84 |
| Urban | 41704 | 81.23 | 38126 | 85.13 | 19706 | 82.16 |
| **Number of Chronic Conditions** |  |  |  |  |  |  |
| 0 | 10288 | 20.86 | 5246 | 12.18 | 2794 | 12.06 |
| 1 | 12733 | 25.81 | 8560 | 19.87 | 4775 | 20.61 |
| 2 | 10686 | 21.66 | 8970 | 20.82 | 5064 | 21.86 |
| 3+ | 15618 | 31.66 | 20308 | 47.14 | 10532 | 45.47 |
| **Type of Alcohol Drinker** |  |  |  |  |  |  |
| Non-drinkers during last 12 months | 7644 | 14.90 | 6007 | 13.42 | 4350 | 18.18 |
| Binge drinker | 2691 | 5.24 | 1996 | 4.46 | 1818 | 7.60 |
| Regular/Occasional drinker | 40974 | 79.81 | 36752 | 82.12 | 17760 | 73.65 |
| **Type of Smoker** |  |  |  |  |  |  |
| Current smoker | 4843 | 9.49 | 3285 | 7.37 | 1448 | 6.11 |
| Former smoker | 30530 | 59.80 | 27313 | 61.3 | 14729 | 62.15 |
| Never smoked | 15684 | 30.72 | 13961 | 31.33 | 7523 | 31.74 |

^1^An average of 7 years passed between CLSA baseline and the COVID-19 exit interview.

Additional File 1b. Comparison of sociodemographic and health characteristics from CLSA Follow-up1 for participants who completed the COVID-19 Exit questionnaire (n=24,114) and participants who did not complete the COVID-19 Exit questionnaire (n=18,343).

|  | **Completed**  **COVID-19 EXIT (n=24,114)** | | **Did not Complete COVID-19 Exit Survey**  **(n=18,343)** | | **Standardized Mean Difference** |
| --- | --- | --- | --- | --- | --- |
|  | **n** | **%** | **n** | **%** |  |
| **Age Group** |  |  |  |  |  |
| <55 | 3,155 | 13.16 | 3,259 | 18.20 | -0.139 |
| 55-64 | 8,314 | 34.68 | 5,919 | 33.06 | 0.034 |
| 65-74 | 7,804 | 32.55 | 4,678 | 26.13 | 0.141 |
| 75+ | 4,701 | 19.61 | 4,047 | 22.61 | -0.073 |
|  |  |  |  |  |  |
| **Sex** |  |  |  |  |  |
| Female | 12,819 | 53.16 | 8,960 | 48.85 | 0.086 |
| Male | 11,295 | 46.84 | 9,383 | 51.15 | -0.086 |
| **Ethnicity** |  |  |  |  |  |
| European | 22,439 | 93.79 | 16,612 | 91.58 | 0.085 |
| Non-European | 1,485 | 6.21 | 1,527 | 8.42 | -0.085 |
| **Annual Household Income** |  |  |  |  |  |
| Less than $20,000 | 861 | 3.80 | 988 | 5.98 | -0.101 |
| $20,000-$49,999 | 4,855 | 21.45 | 4,223 | 25.56 | -0.097 |
| $50,000-$99,999 | 8,571 | 37.87 | 5,702 | 34.51 | 0.070 |
| $100,000-$149,999 | 4,589 | 20.27 | 2,933 | 17.75 | 0.075 |
| $150,000 or more | 3,758 | 16.6 | 2,676 | 16.2 | 0.011 |
| **Number of People Living in the Same Household** |  |  |  |  |  |
| Living alone | 5,599 | 23.41 | 4,261 | 24.04 | -0.015 |
| Not living alone | 18,323 | 76.59 | 13,460 | 75.96 | 0.015 |
| **Living Area** |  |  |  |  |  |
| Rural | 3,309 | 13.81 | 2,926 | 16.36 | -0.071 |
| Urban | 20,649 | 86.19 | 14,964 | 83.64 | 0.071 |
| **Number of Chronic Conditions** |  |  |  |  |  |
| 0 | 2,794 | 12.06 | 2,318 | 13.41 | -0.041 |
| 1 | 4,775 | 20.61 | 3,468 | 20.07 | 0.014 |
| 2 | 5,064 | 21.86 | 3,524 | 20.39 | 0.036 |
| 3+ | 10,532 | 45.47 | 7,970 | 46.12 | -0.013 |
| **Type of Alcohol Drinker** |  |  |  |  |  |
| Non-drinkers during last 12 months | 2,777 | 11.6 | 2,586 | 14.47 | -0.085 |
| Binge drinker | 968 | 4.04 | 926 | 5.18 | -0.054 |
| Regular/Occasional drinker | 20,202 | 84.36 | 14,364 | 80.35 | 0.105 |
| **Type of Smoker** |  |  |  |  |  |
| Current smoker | 1,387 | 5.82% | 1837 | 8.81 | -0.119 |
| Former smoker | 14,787 | 62.05% | 12584 | 60.33 | 0.041 |
| Never smoked | 7,656 | 32.13% | 6438 | 30.86 | 0.024 |
