# Supplementary material for: Increased prevalence of loneliness and associated risk factors during the COVID-19 pandemic: findings from the Canadian Longitudinal Study on Aging (CLSA)
Source: BMC Public Health. 2023 May 12;23:872. doi: 10.1186/s12889-023-15807-4 (PMC10175060; doi:10.1186/s12889-023-15807-4)
Supplement: Supplementary file 2 — Additional file 2. a: Predictors of loneliness during the COVID-19 pandemic, adjusted for pre-pandemic loneliness and participant characteristics in the pre-pandemic period using an alternative cut-off value “≥4”. b: Predictors of loneliness during the COVID-19 pandemic, adjusted for pre-pandemic loneliness and participant characteristics in the pre-pandemic period using an alternative cut-off value “≥6”. c: Predictors of loneliness during the COVID-19 pandemic, adjusted for pre-pandemic loneliness and participant characteristics in the pre-pandemic period using lagged linear regression. d: Predictors of ln(loneliness) during the COVID-19 pandemic, adjusted for pre-pandemic ln(loneliness) and participant characteristics in the pre-pandemic period using lagged linear regression. [file 12889_2023_15807_MOESM2_ESM.docx]

Additional File 2a: Predictors of loneliness during the COVID-19 pandemic, adjusted for pre-pandemic loneliness and participant characteristics in the pre-pandemic period using an alternative cut-off value “≥4”

| OUTCOME: LONELINESS at COVID Exit | Model 1  (n=23,544)  OR (95% CIs) | Model 6  (n=20,033)  OR (95% CIs) |
| --- | --- | --- |
| **Loneliness at FU1 (ref="Not lonely")** |  |  |
| Lonely | **4.85 (4.57,5.15)** | **4.13 (3.86,4.42)** |
| **Age (ref="75+")** |  |  |
| <55 | - | **1.21 (1.06,1.39)** |
| 55-64 | - | **1.15 (1.04,1.27)** |
| 65-74 | - | **1.12 (1.02,1.22)** |
| **Sex (ref="Male")** |  |  |
| Female | - | **1.24 (1.16,1.32)** |
| **Ethnicity (ref="European")** |  |  |
| Non-European | - | 0.91 (0.8,1.04) |
| **Education (ref="Post-secondary education/degree/diploma")** |  |  |
| Secondary school graduation, no post-secondary education | - | **0.73 (0.62,0.86)** |
| Less than secondary school graduation | - | **0.89 (0.8,0.99)** |
| **HH Income (ref="$150,000 or more")** |  |  |
| Less than $20,000 | - | 0.93 (0.76,1.15) |
| $20,000 or more, but less than $50,000 | - | 1.03 (0.91,1.15) |
| $50,000 or more, but less than $100,000 | - | 1.02 (0.93,1.12) |
| $100,000 or more, but less than $150,000 | - | 1.06 (0.96,1.17) |
| **Number of people living in the same HH (ref="Not living alone")** |  |  |
| Living alone | - | **1.26 (1.15,1.38)** |
| **Dwelling Type (ref="House (single detached, semi-detached, duplex or townhouse)")** |  |  |
| Apartment or condominium | - | 1.09 (0.99,1.19) |
| Other | - | 0.97 (0.77,1.23) |
| **Living Area (ref="Rural")** |  |  |
| Urban | - | **1.12 (1.02,1.22)** |
| **Province (ref="NS")** |  |  |
| AB | - | **1.67 (1.44,1.93)** |
| BC | - | **1.56 (1.37,1.78)** |
| MB | - | **1.83 (1.57,2.12)** |
| NB | - | 1.05 (0.82,1.34) |
| NL | - | 1.06 (0.9,1.25) |
| ON | - | **1.65 (1.45,1.87)** |
| PEI | - | 0.93 (0.71,1.22) |
| QC | - | **1.35 (1.19,1.54)** |
| SK | - | **1.58 (1.26,1.99)** |
| **Depression (ref="Negative screen for depression")** |  |  |
| Positive screen for depression | - | **2.14 (1.91,2.38)** |
| **Number of CCs (ref="0")** |  |  |
| 1 | - | 1.1 (0.99,1.23) |
| 2 | - | **1.17 (1.05,1.3)** |
| 3+ | - | **1.33 (1.2,1.48)** |
| **Type of Alcohol Drinker (ref="Did not drink in the last 12 months")** |  |  |
| Binge drinker | - | 1.12 (0.94,1.34) |
| Regular drinker | - | **1.24 (1.12,1.37)** |
| Occasional drinker | - | **1.2 (1.05,1.36)** |
| **Type of Smoker (ref="Never smoked")** |  |  |
| Current smoker | - | 1.06 (0.92,1.23) |
| Former smoker | - | **1.07 (1.01,1.15)** |
| **PASE (ref="low risk")** |  |  |
| At risk | - | 0.99 (0.93,1.06) |
| **Social Participation (ref="High social participation")** |  |  |
| Low social participation | - | 1 (0.92,1.09) |
| **CHANGE in average weekday alone time during the day (ref="Same")** |  |  |
| Less than usual | - | **1.26 (1.14,1.39)** |
| More than usual | - | 1.07 (0.98,1.18) |
| **CHANGE in average weekend alone time during the day (ref="Same")** |  |  |
| Less than usual | - | **1.24 (1.12,1.37)** |
| More than usual | - | 0.99 (0.9,1.1) |
| **Essential Worker (ref="Not works outside of residence")** |  |  |
| Yes | - | **0.73 (0.66,0.82)** |
| No | - | 1 (0.91,1.1) |

Sex, ethnicity, and education come from CLSA baseline; all other variables come from FUP1 sample except for change in average weekday alone time during the day, change in average weekend alone time during the day, essential worker which come from COVID baseline sample.

Physical Activity: low risk = at least 150 minutes of moderate-intensity or at least 75 minutes of vigorous-intensity physical activity per week; high risk = less than 150 minutes of moderate-intensity or at least 75 minutes of vigorous-intensity physical activity per week.

Social Participation: low social participation = in the lowest sex- and age-specific quintiles of social participation; high social participation = in the top four sex- and age-specific quintiles of social participation

^1^ Three individuals who lived in Yukon and Nunavut at the time of the COVID-19 exit interview were added to the missing category and excluded from analyses.

Additional File 2b: Predictors of loneliness during the COVID-19 pandemic, adjusted for pre-pandemic loneliness and participant characteristics in the pre-pandemic period using an alternative cut-off value “≥6”

| OUTCOME: LONELINESS at COVID Exit | Model 1  (n=23,519)  OR (95% CIs) | Model 6  (n=20,005)  OR (95% CIs) |
| --- | --- | --- |
| **Loneliness at FU1 (ref="Not lonely")** |  |  |
| Lonely | **7.57 (6.93,8.27)** | **5.4 (4.86,6.01)** |
| **Age (ref="75+")** |  |  |
| <55 | - | **1.4 (1.19,1.65)** |
| 55-64 | - | **1.3 (1.16,1.47)** |
| 65-74 | - | **1.13 (1.01,1.26)** |
| **Sex (ref="Male")** |  |  |
| Female | - | **1.36 (1.25,1.47)** |
| **Ethnicity (ref="European")** |  |  |
| Non-European | - | 1 (0.86,1.16) |
| **Education (ref="Post-secondary education/degree/diploma")** |  |  |
| Secondary school graduation, no post-secondary education | - | **0.74 (0.61,0.9)** |
| Less than secondary school graduation | - | **0.94 (0.83,1.07)** |
| **HH Income (ref="$150,000 or more")** |  |  |
| Less than $20,000 | - | 1.05 (0.83,1.32) |
| $20,000 or more, but less than $50,000 | - | 1.19 (1.03,1.37) |
| $50,000 or more, but less than $100,000 | - | 1.22 (1.08,1.37) |
| $100,000 or more, but less than $150,000 | - | 1.11 (0.98,1.27) |
| **Number of people living in the same HH (ref="Not living alone")** |  |  |
| Living alone | - | **1.43 (1.3,1.58)** |
| **Dwelling Type (ref="House (single detached, semi-detached, duplex or townhouse)")** |  |  |
| Apartment or condominium | - | 1 (0.9,1.11) |
| Other | - | 1 (0.77,1.29) |
| **Living Area (ref="Rural")** |  |  |
| Urban | - | **1.2 (1.07,1.34)** |
| **Province (ref="NS")** |  |  |
| AB | - | **1.59 (1.33,1.91)** |
| BC | - | **1.44 (1.22,1.7)** |
| MB | - | **1.77 (1.47,2.13)** |
| NB | - | 1.04 (0.75,1.44) |
| NL | - | 1.03 (0.83,1.29) |
| ON | - | **1.47 (1.25,1.72)** |
| PEI | - | 1.2 (0.85,1.7) |
| QC | - | **1.26 (1.07,1.49)** |
| SK | - | **1.58 (1.21,2.08)** |
| **Depression (ref="Negative screen for depression")** |  |  |
| Positive screen for depression | - | **2.27 (2.06,2.51)** |
| **Number of CCs (ref="0")** |  |  |
| 1 | - | 1.1 (0.96,1.27) |
| 2 | - | **1.24 (1.08,1.43)** |
| 3+ | - | **1.44 (1.26,1.65)** |
| **Type of Alcohol Drinker (ref="Did not drink in the last 12 months")** |  |  |
| Binge drinker | - | 0.91 (0.73,1.14) |
| Regular drinker | - | **1.08 (0.96,1.21)** |
| Occasional drinker | - | **1.08 (0.93,1.26)** |
| **Type of Smoker (ref="Never smoked")** |  |  |
| Current smoker | - | 1.1 (0.93,1.29) |
| Former smoker | - | **1.05 (0.97,1.14)** |
| **PASE (ref="low risk")** |  |  |
| At risk | - | 1.03 (0.95,1.12) |
| **Social Participation (ref="High social participation")** |  |  |
| Low social participation | - | 1.11 (1.01,1.22) |
| **CHANGE in average weekday alone time during the day (ref="Same")** |  |  |
| Less than usual | - | **1.34 (1.2,1.49)** |
| More than usual | - | 0.96 (0.85,1.08) |
| **CHANGE in average weekend alone time during the day (ref="Same")** |  |  |
| Less than usual | - | **1.29 (1.15,1.45)** |
| More than usual | - | 1.01 (0.89,1.15) |
| **Essential Worker (ref="Not works outside of residence")** |  |  |
| Yes | - | **0.87 (0.76,1)** |
| No | - | 1.01 (0.9,1.14) |

Sex, ethnicity, and education come from CLSA baseline; all other variables come from FUP1 sample except for change in average weekday alone time during the day, change in average weekend alone time during the day, essential worker which come from COVID baseline sample.

Physical Activity: low risk = at least 150 minutes of moderate-intensity or at least 75 minutes of vigorous-intensity physical activity per week; high risk = less than 150 minutes of moderate-intensity or at least 75 minutes of vigorous-intensity physical activity per week.

Social Participation: low social participation = in the lowest sex- and age-specific quintiles of social participation; high social participation = in the top four sex- and age-specific quintiles of social participation

^1^ Three individuals who lived in Yukon and Nunavut at the time of the COVID-19 exit interview were added to the missing category and excluded from analyses.

Additional File 2c: Predictors of loneliness during the COVID-19 pandemic, adjusted for pre-pandemic loneliness and participant characteristics in the pre-pandemic period using lagged linear regression.

| OUTCOME: LONELINESS at COVID Exit | Model 1  (n=23,398)  Beta (P-value) | Model 6  (n=19,925)  Beta (P-value) |
| --- | --- | --- |
| **Loneliness at FUP1** | **0.591 (<.0001)** | **0.516 (<.0001)** |
| **Age (ref="75+”)** |  |  |
| <55 | - | **0.153 (0.0003)** |
| 55-64 | - | **0.139 (<.0001)** |
| 65-74 | - | **0.089 (0.0015)** |
| **Sex (ref="Male")** |  |  |
| Female | - | **0.181 (<.0001)** |
| **Ethnicity (ref="European")** |  |  |
| Non-European | - | -0.023 (0.5618) |
| **Education (ref="Post-secondary education/degree/diploma”)** |  |  |
| Secondary school graduation, no post-secondary education | - | **-0.21 (<.0001)** |
| Less than secondary school graduation | - | -0.046 (0.1647) |
| **HH Income (ref="$150,000 or more”)** |  |  |
| <$20,000 | - | -0.106 (0.0878) |
| $20,000-$49,999 | - | 0.01 (0.7756) |
| $50,000-$99,999 | - | 0.026 (0.3839) |
| $100,000-$149,999 | - | 0.039 (0.2134) |
| **Number of people living in the same HH (ref="Not living alone”)** |  |  |
| Living alone | - | 0.171 (<.0001) |
| **Dwelling Type (ref="House (single detached, semi-detached, duplex or townhouse)”)** |  |  |
| Apartment or condominium | - | 0.011 (0.6926) |
| Other | - | 0.016 (0.8176) |
| **Living Area (ref="Rural”)** |  |  |
| Urban | - | **0.096 (0.0007)** |
| **Province (ref="NS”)** |  |  |
| AB | - | **0.265 (<.0001)** |
| BC | - | **0.236 (<.0001)** |
| MB | - | **0.362 (<.0001)** |
| NB | - | 0.069 (0.3629) |
| NL | - | -0.002 (0.9723) |
| ON | - | **0.281 (<.0001)** |
| PEI | - | 0.01 (0.9081) |
| QC | - | **0.158 (<.0001)** |
| SK | - | **0.257 (0.0003)** |
| **Depression (ref="Negative screen for depression”)** |  |  |
| Positive screen for depression | - | **0.448 (<.0001)** |
| **Number of CCs (ref="0")** |  |  |
| 1 | - | 0.042 (0.2207) |
| 2 | - | **0.121 (0.0004)** |
| 3+ | - | **0.208 (<.0001)** |
| **Type of Alcohol Drinker (ref="Did not drink in the last 12 months”)** |  |  |
| Binge drinker | - | -0.008 (0.8835) |
| Regular drinker | - | **0.096 (0.0019)** |
| Occasional drinker | - | 0.056 (0.1543) |
| **Type of Smoker (ref="Never smoked”)** |  |  |
| Current smoker | - | **0.102 (0.0192)** |
| Former smoker | - | **0.054 (0.0091)** |
| **PASE (ref="low risk”)** |  |  |
| At risk | - | 0.018 (0.3926) |
| **Social Participation (ref="High social participation”)** |  |  |
| Low social participation | - | 0.009 (0.7162) |
| **CHANGE in average weekday alone time during the day (ref="Same”)** |  |  |
| Less than usual | - | **0.171 (<.0001)** |
| More than usual | - | 0.017 (0.5818) |
| **CHANGE in average weekend alone time during the day (ref="Same”)** |  |  |
| Less than usual | - | **0.18 (<.0001)** |
| More than usual | - | -0.013 (0.6722) |
| **Essential Worker (ref="Not works outside of residence”)** |  |  |
| Yes | - | **-0.138 (<.0001)** |
| No | - | 0.01 (0.744) |

Sex, ethnicity, and education come from CLSA baseline; all other variables come from FUP1 sample except for change in average weekday alone time during the day, change in average weekend alone time during the day, essential worker which come from COVID baseline sample.

Physical Activity: low risk = at least 150 minutes of moderate-intensity or at least 75 minutes of vigorous-intensity physical activity per week; high risk = less than 150 minutes of moderate-intensity or at least 75 minutes of vigorous-intensity physical activity per week.

Social Participation: low social participation = in the lowest sex- and age-specific quintiles of social participation; high social participation = in the top four sex- and age-specific quintiles of social participation

^1^ Three individuals who lived in Yukon and Nunavut at the time of the COVID-19 exit interview were added to the missing category and excluded from analyses.

Additional File 2d: Predictors of ln(loneliness) during the COVID-19 pandemic, adjusted for pre-pandemic ln(loneliness) and participant characteristics in the pre-pandemic period using lagged linear regression.

| OUTCOME: LN(LONELINESS) at COVID Exit | Model 1  (n=23,398)  Beta (P value) | Model 6  (n=19,925)  Beta (P value) |
| --- | --- | --- |
| **Ln(Loneliness) at FUP1** | **0.558 (<.0001)** | **0.491 (<.0001)** |
| **Age (ref="75+")** |  |  |
| <55 | - | **0.033 (0.0002)** |
| 55-64 | - | **0.028 (<.0001)** |
| 65-74 | - | **0.019 (0.0016)** |
| **Sex (ref="Male")** |  |  |
| Female | - | **0.039 (<.0001)** |
| **Ethnicity (ref="European")** |  |  |
| Non-European | - | -0.008 (0.3513) |
| **Education (ref="Post-secondary education/degree/diploma")** |  |  |
| Secondary school graduation, no post-secondary education | - | **-0.045 (<.0001)** |
| Less than secondary school graduation | - | -0.011 (0.1016) |
| **HH Income (ref="$150,000 or more")** |  |  |
| <$20,000 | - | -0.021 (0.1107) |
| $20,000-$49,999 | - | 0.001 (0.888) |
| $50,000-$99,999 | - | 0.004 (0.4767) |
| $100,000-$149,999 | - | 0.008 (0.2021) |
| **Number of people living in the same HH (ref="Not living alone")** |  |  |
| Living alone | - | **0.033 (<.0001)** |
| **Dwelling Type (ref="House (single detached, semi-detached, duplex or townhouse)")** |  |  |
| Apartment or condominium | - | 0.004 (0.5039) |
| Other | - | 0.002 (0.9119) |
| **Living Area (ref="Rural")** |  |  |
| Urban | - | **0.02 (0.0007)** |
| **Province (ref="NS")** |  |  |
| AB | - | **0.061 (<.0001)** |
| BC | - | **0.053 (<.0001)** |
| MB | - | **0.081 (<.0001)** |
| NB | - | 0.01 (0.5155) |
| NL | - | 0 (0.9709) |
| ON | - | **0.062 (<.0001)** |
| PEI | - | 0.004 (0.825) |
| QC | - | **0.035 (<.0001)** |
| SK | - | **0.059 (<.0001)** |
| **Depression (ref="Negative screen for depression")** |  |  |
| Positive screen for depression | - | **0.093 (<.0001)** |
| **Number of CCs (ref="0")** |  |  |
| 1 | - | 0.01 (0.1558) |
| 2 | - | **0.025 (0.0006)** |
| 3+ | - | **0.044 (<.0001)** |
| **Type of Alcohol Drinker (ref="Did not drink in the last 12 months")** |  |  |
| Binge drinker | - | 0.002 (0.8354) |
| Regular drinker | - | **0.023 (0.0005)** |
| Occasional drinker | - | 0.015 (0.0779) |
| **Type of Smoker (ref="Never smoked")** |  |  |
| Current smoker | - | **0.019 (0.0447)** |
| Former smoker | - | **0.011 (0.0104)** |
| **PASE (ref="low risk")** |  |  |
| At risk | - | 0.003 (0.5723) |
| **Social Participation (ref="High social participation")** |  |  |
| Low social participation | - | 0 (0.9705) |
| **CHANGE in average weekday alone time during the day (ref="Same")** |  |  |
| Less than usual | - | **0.036 (<.0001)** |
| More than usual | - | 0.006 (0.374) |
| **CHANGE in average weekend alone time during the day (ref="Same")** |  |  |
| Less than usual | - | **0.038 (<.0001)** |
| More than usual | - | -0.003 (0.7042) |
| **Essential Worker (ref="Not works outside of residence")** |  |  |
| Yes | - | **-0.034 (<.0001)** |
| No | - | 0.001 (0.8369) |

Sex, ethnicity, and education come from CLSA baseline; all other variables come from FUP1 sample except for change in average weekday alone time during the day, change in average weekend alone time during the day, essential worker which come from COVID baseline sample.

Physical Activity: low risk = at least 150 minutes of moderate-intensity or at least 75 minutes of vigorous-intensity physical activity per week; high risk = less than 150 minutes of moderate-intensity or at least 75 minutes of vigorous-intensity physical activity per week.

Social Participation: low social participation = in the lowest sex- and age-specific quintiles of social participation; high social participation = in the top four sex- and age-specific quintiles of social participation

^1^ Three individuals who lived in Yukon and Nunavut at the time of the COVID-19 exit interview were added to the missing category and excluded from analyses.
